# Quality of Life Outcomes Following Total Temporomandibular Joint Replacement: A Systematic Review of Long-Term Efficacy, Functional Improvements, and Complication Rates Across Prosthesis Types

**DOI:** 10.3390/jcm14144859

**Published:** 2025-07-09

**Authors:** Luis Eduardo Almeida, Samuel Zammuto, Louis G. Mercuri

**Affiliations:** 1 Surgical Sciences Department, Oral and Maxillofacial, School of Dentistry, Marquette University, Milwaukee, WI 53233, USA; 2 Department of Orthopaedic Surgery, Rush University Medical Center, 1611 W. Harrison St. Suite 204, Chicago, IL 60612, USA

**Keywords:** total temporomandibular joint replacement, temporomandibular joint, patient quality of life

## Abstract

**Introduction:** Total temporomandibular joint replacement (TMJR) is a well-established surgical solution for patients with severe TMJ disorders. It aims to relieve chronic pain, restore jaw mobility, and significantly enhance quality of life. This systematic review evaluates QoL outcomes following TMJR, analyzes complication profiles, compares custom versus stock prostheses, explores pediatric applications, and highlights technological innovations shaping the future of TMJ reconstruction. **Methods:** A systematic search of PubMed, Embase, and the Cochrane Library was conducted throughout April 2025 in accordance with PRISMA 2020 guidelines. Sixty-four studies were included, comprising 2387 patients. **Results:** Primary outcomes assessed were QoL improvement, pain reduction, and functional gains such as maximum interincisal opening (MIO). Secondary outcomes included complication rates and technological integration. TMJR consistently led to significant pain reduction (75–87%), average MIO increases of 26–36 mm, and measurable QoL improvements across physical, social, and psychological domains. Custom prostheses were particularly beneficial in anatomically complex or revision cases, while stock devices generally performed well for standard anatomical conditions. Pediatric TMJR demonstrated functional and airway benefits with no clear evidence of growth inhibition over short- to medium-term follow-up. Complications such as heterotopic ossification (~20%, reduced to <5% with fat grafting), infection (3–4.9%), and chronic postoperative pain (~20–30%) were reported but were largely preventable or manageable. Recent advancements, including CAD/CAM planning, 3D-printed prostheses, augmented-reality-assisted surgery, and biofilm-resistant materials, are enhancing personalization, precision, and implant longevity. **Conclusions:** TMJR is a safe and transformative treatment that consistently improves QoL in patients with end-stage TMJ disease. Future directions include long-term registry tracking, growth-accommodating prosthesis design, and biologically integrated smart implants.

## 1. Introduction

Disorders of the temporomandibular joint (TMJ) greatly impact patients’ daily lives, often leading to persistent facial pain, limited jaw movement, difficulties with speaking or chewing, and emotional strain. Etiologies include osteoarthritis, trauma, ankylosis, rheumatoid arthritis, juvenile idiopathic arthritis (JIA), idiopathic condylar resorption, and congenital deformities. In severe cases, joint degeneration leads to airway compromise, malnutrition, and functional disability [1,2,3,4].

Historically, end-stage TMJ pathology was managed with autogenous reconstructions such as costochondral grafts. However, early alloplastic implants like Proplast-Teflon were associated with high failure rates, foreign-body reactions, and mechanical degradation [3,5]. These challenges led to the evolution of modern total temporomandibular joint replacement (TMJR) systems, including both FDA-approved stock prostheses (e.g., Biomet/Lorenz) and custom-fabricated prostheses (e.g., TMJ Concepts, Melbourne 3D-printed system) [2,6].

TMJR has become the preferred surgical treatment for patients with end-stage TMJ disease that does not respond to conservative management. A wide body of evidence supports its effectiveness in reducing pain, increasing jaw mobility, and improving essential functions such as chewing, speech, and quality of life. These functional improvements contribute to better nutrition, emotional well-being, and long-term QoL [7,8,9,10,11,12].

TMJR, while successful, carries risks. Heterotopic ossification (HO) occurs in 15–20% of cases without fat grafting but drops below 5% with prophylactic use of autologous fat [13,14]. Prosthetic joint infections are uncommon (3–4.9%) but can lead to prosthesis failure and necessitate staged revision [15,16]. Chronic postoperative pain affects 20–30% of patients, often linked to central sensitization or comorbid muscular disorders [17,18].

A critical decision in TMJR planning is choosing between stock and custom prostheses. Custom devices, derived from CT imaging via CAD/CAM, offer superior anatomical fit and are ideal for complex or revision cases [2,6,18]. Stock prostheses are readily available and lower in cost, providing comparable outcomes in patients with standard anatomy [8,19,20,21,22]. Meta-analyses have found no significant difference in overall pain relief or QoL outcomes between stock and custom designs when matched to appropriate cases [20,23,24].

Recent technological advances are rapidly improving TMJR precision and outcomes. CAD/CAM planning, virtual surgical simulation, and 3D printing allow for enhanced prosthesis customization [19,25,26]. Augmented reality (AR)–assisted navigation offers real-time visualization during surgery [27,28]. Meanwhile, innovations in biofilm-resistant coatings and smart implants are helping to reduce infection risk and monitor prosthesis health [5,26,29]. Tissue-engineered solutions, such as 3D-bioprinted condylar scaffolds, represent a future frontier in biologic TMJ reconstruction [29,30].

Historically, pediatric TMJR was avoided due to concerns over facial growth inhibition. However, recent studies have shown that in carefully selected skeletally immature patients, TMJR leads to substantial improvements in function, airway patency, and QoL—without short-term evidence of growth arrest [11,22,31,32]. Long-term surveillance remains essential, and the development of expandable or growth-accommodating prostheses is a key area of ongoing research [31,33,34,35].

Therefore, the objective of this systematic review is to evaluate the long-term quality of life (QoL) outcomes following total temporomandibular joint replacement (TMJR), with a focus on functional improvements, complication profiles, and differences between custom and stock prostheses across adult and pediatric populations.

## 2. Materials and Methods

### 2.1. Study Design

Although this review was not registered in PROSPERO, all steps—including objectives, eligibility criteria, search strategy, and synthesis plan—were pre-defined and followed to ensure transparency and reproducibility. This limitation is acknowledged in the manuscript.

### 2.2. Eligibility Criteria

Eligibility was defined using the PICOS framework:

- Population: Patients of any age undergoing total temporomandibular joint replacement (TMJR) for end-stage TMJ pathology, including osteoarthritis, rheumatoid arthritis, juvenile idiopathic arthritis, trauma, ankylosis, condylar resorption, or neoplasms.
- Intervention: Alloplastic TMJR using stock or custom prostheses. Studies including adjunctive procedures (e.g., fat grafting, orthognathic surgery) were included if TMJR outcomes were clearly delineated.
- Comparator: Studies with or without comparator groups were eligible. Comparisons could include pre- vs. postoperative outcomes, stock vs. custom devices, or TMJR vs. other surgical options.
- Outcomes:
  ○ Primary outcomes: Pain reduction (e.g., VAS scores), functional recovery (e.g., maximum interincisal opening [MIO]), and quality of life (e.g., SF-36, TMJ-specific PROMs, narrative assessments).
  ○ Secondary outcomes: Complication rates (e.g., heterotopic ossification, infection, prosthesis failure, persistent pain), long-term prosthesis survival, and the integration of advanced technologies (e.g., CAD/CAM, 3D printing, AR).
- Study Design: Randomized controlled trials (RCTs), prospective and retrospective cohort studies, and case series including ≥5 patients were included.

Exclusion Criteria:

- Case reports or series with <5 patients;
- In vitro or biomechanical-only studies;
- Editorials, reviews, commentaries, or abstracts without peer-reviewed data;
- Non-English language studies without available translations.

### 2.3. Search Strategy

Three databases were systematically searched from inception to 30 April 2025:

- PubMed (MEDLINE);
- Embase;
- Cochrane Library.

Search terms included combinations of MeSH and free-text keywords:

(“temporomandibular joint replacement” OR “TMJ prosthesis” OR “TMJR” OR “total joint replacement”) AND

(“quality of life” OR “pain” OR “function” OR “MIO” OR “complications” OR “prosthesis type” OR “CAD/CAM” OR “3D printing” OR “pediatrics” OR “technology”)

Filters were applied to include only human studies published in English.

Additionally, reference lists of included articles and relevant reviews were screened manually to identify further eligible studies.

### 2.4. Study Selection

**Figure 1:**
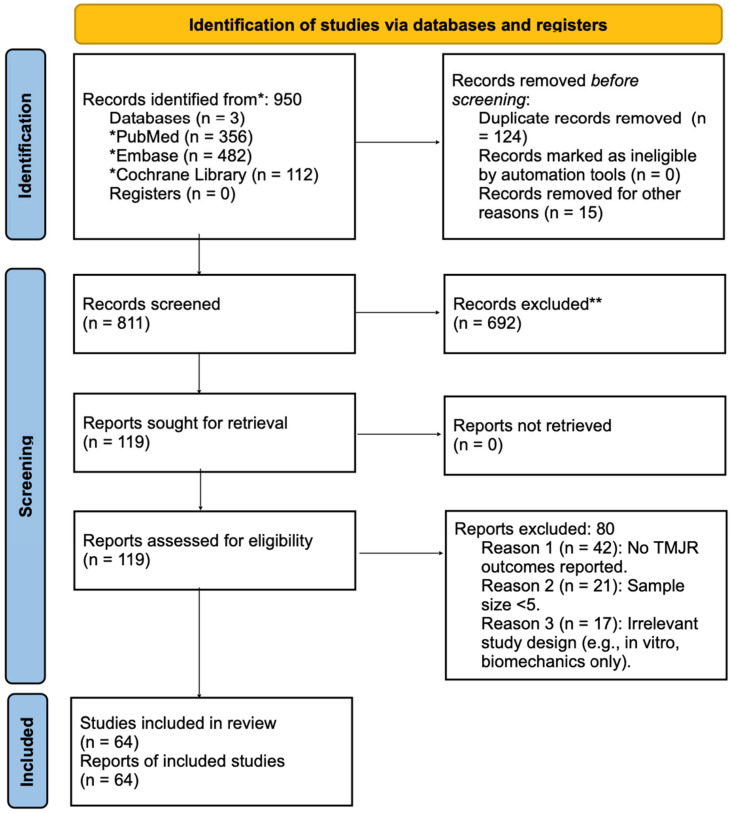
PRISMA 2020 flow diagram. * Studies retrieved for studies meeting inclusion. ** Studies that were excluded after screening.

Two reviewers independently screened all titles and abstracts. Full-text articles were retrieved for studies meeting inclusion criteria or requiring clarification. Disagreements were resolved by discussion or third-party adjudication. The study selection process is depicted in the PRISMA 2020 flow diagram (Figure 1).

### 2.5. Data Extraction

A standardized extraction form was used by two independent reviewers to collect the following:

- Study characteristics (authors, year, country, design);
- Sample size and demographics;
- Indications for TMJR;
- Prosthesis type (stock vs. custom; manufacturer if available);
- Surgical adjuncts (e.g., fat grafting, VSP):
  ○ Follow-up duration;
  ○ Outcome measures;
  ○ Pain scores (pre- and post-op);
  ○ MIO (pre- and post-op);
  ○ QoL measures (validated instruments or narrative satisfaction).

Complications (type and rate);
Use of technology (e.g., CAD/CAM, 3D printing, AR, tissue engineering);
Discrepancies in data extraction were resolved through consensus.

### 2.6. Risk of Bias Assessment

- Randomized controlled trials were assessed using the Cochrane Risk of Bias 2 (RoB 2) tool (11-13 Cavendish Square, London, W1G 0AN, United Kingdom)
- Observational studies were evaluated using the Newcastle–Ottawa Scale (NOS).

Studies were categorized as having low, moderate, or high risk of bias based on selection, comparability, and outcome assessment. Bias ratings were considered in the synthesis.

### 2.7. Data Synthesis and Analysis

1. Due to heterogeneity in study design, outcome measures, and follow-up durations, meta-analysis was not feasible. A qualitative synthesis was performed.
2. Findings were organized under the following domains:
3. Pain reduction;
4. Functional recovery (MIO, diet);
5. Quality of life outcomes;
6. Complications and management;
7. Prosthesis type comparison (custom vs. stock);
8. Pediatric TMJR outcomes;
9. Technological innovations (CAD/CAM, AR, 3D printing, tissue engineering).

Data was summarized in tables and narrative text where appropriate.

### 2.8. Ethical Considerations

This review involved no new human or animal data. All information is derived from previously published, peer-reviewed studies. As such, institutional review board (IRB) approval was not required.

## 3. Results

### 3.1. Study Selection

- The database search identified a total of 950 records: PubMed (n = 356), Embase (n = 482), and Cochrane Library (n = 112). After removing 124 duplicates and 15 non-relevant records (e.g., non-human studies, editorials), 811 records were screened by title and abstract. Of these, 692 were excluded based on the predefined eligibility criteria.
- Full-text review was conducted on 119 studies, with 80 excluded for the following reasons:

No reported TMJR outcomes (n = 42);

Sample size fewer than 5 patients (n = 21);

In vitro or non-clinical/technical studies (n = 17).

An additional 25 studies were identified through manual reference screening and citation tracking.

A total of 64 studies met all inclusion criteria and were included in the qualitative synthesis.

- No reported TMJR outcomes (n = 42);
- Sample size fewer than 5 patients (n = 21);
- In vitro or non-clinical/technical studies (n = 17);
- A total of 64 studies were included in the final qualitative synthesis: 39 studies from database screening and an additional 25 identified through manual reference screening and citation tracking.

### 3.2. Study Characteristics

The 64 studies included were published between 2002 and 2024, with sample sizes ranging from 5 to 1262 patients (Table 1). Cohort characteristics are summarized as follows:

- Age range: 13–72 years;
- Gender: Slight female predominance;
- Prosthesis types: 38 custom-fabricated, 19 stock, 8 hybrid or semi-custom;
- Follow-up periods: 6 months to over 20 years;
- Adjunctive procedures reported: fat grafting, orthognathic surgery, virtual surgical planning (VSP), CAD/CAM-guided surgery, and augmented reality-assisted navigation.

### 3.3. Quality of Life Outcomes 

Across nearly all included studies, TMJR resulted in significant improvements in patient-reported quality of life (QoL). These were assessed using:

- The SF-36 Health Survey;
- TMJ-specific patient-reported outcome measures (PROMs);
- Narrative satisfaction reports or structured interviews.
- Reported QoL benefits included:
- Reduced pain and disability;
- Improved chewing, speech, and diet;
- Enhanced social confidence, sleep quality, and emotional well-being.
- Notable studies:
- Gupta et al. reported significant SF-36 improvements across pain, vitality, and social domains [7];
- Alba et al. observed airway, speech, and psychosocial gains in pediatric patients [11];
- Balel and Mercuri documented reduced somatization and anxiety [12].

### 3.4. Functional Outcomes and Pain Reduction

Maximum Interincisal Opening (MIO):

- Preoperative range: 8–25 mm;
- Postoperative range: 30–42 mm;
- Mean gain: 26–36 mm.

Sustained improvements were observed in ankylosis and complex TMJ cases, including pediatric patients [6,9,15,23,24,36,37,38].

Pain Reduction:

Most studies reported a 75–87% reduction in VAS scores.

- Consistent across unilateral and bilateral TMJR;
- Comparable between custom and stock prosthesis groups [7,8,9,15,20,30,39,40,41,42,43,44].

### 3.5. Complications and Management (Table 1)

- While TMJR is generally safe, reported complications included the following:

**Table 1:** Complications in TMJR.

Complication	Approximate Rate	Notes
Heterotopic ossification (HO)	~20% (without fat graft); <5% (with fat graft)	Prevented using autologous fat interposition [13,14]
Prosthetic infection	3–4.9%	Managed with antibiotics, debridement, or staged revision [15,16]
Mechanical failure/ loosening	~4–5%	Often seen in long-term follow-up; usually revised to custom [16,22,24,45]
Chronic pain	20–30%	Frequently due to central sensitization; treated with multimodal pain therapy [17,34]
Facial nerve injury	~10% transient; ~1–2% permanent	Minimized with careful dissection and nerve monitoring [46]

### 3.6. Stock vs. Custom Prostheses

- Custom prostheses demonstrated slightly better MIO recovery and anatomic fit in complex or revision cases [2,6,18,19,47,48,49,50,51,52,53];
- Stock prostheses were comparable in pain reduction, QoL outcomes, and overall satisfaction in routine cases [8,20,21,22,54,55,56];
- Meta-analyses and cohort comparisons found no statistically significant difference in functional outcomes when prosthesis selection was appropriate [20,23,24].

### 3.7. Pediatric TMJR

Pediatric patients undergoing TMJR experienced:

- Significant gains in function, chewing, speech, and psychosocial well-being [11,22,31,32];
- No clear evidence of mandibular growth inhibition during 2–8 years of follow-up;
- Ongoing monitoring is needed as some patients may require revision during growth;
- Development of expandable prostheses remains a critical research priority [33,35].

### 3.8. Technological Innovations

- Emerging technologies improving TMJR include the following:
- CAD/CAM and 3D printing—enhance implant fit, reduce intraoperative modifications [19,25,26,35,57,58];
- Augmented reality (AR)—assists with intraoperative alignment and surgical precision [19,28,59];
- Biofilm-resistant materials—silver-ion coatings and antibiotic-eluting surfaces to reduce infection risk [5,29,60,61];
- Smart prosthetics—force-sensing implants capable of detecting mechanical wear in real time [26,61,62];
- Tissue engineering—preclinical development of 3D-bioprinted condyles and biologic scaffolds [29,30].

These innovations are steadily transforming TMJR into a more personalized, durable, and biologically integrated treatment modality.

## 4. Discussion

### 4.1. Interpretation of Findings

This systematic review demonstrates that total temporomandibular joint replacement (TMJR) is a highly effective, safe, and durable treatment for end-stage TMJ disorders. Across varied populations and indications—including osteoarthritis, inflammatory arthritis, ankylosis, trauma, and idiopathic condylar resorption—TMJR consistently provides substantial pain relief, functional restoration, and improved quality of life (QoL) [6,7,8,9,10,15,22,29]. Reported pain reductions range from 75 to 87%, while maximum interincisal opening (MIO) improvements average 26–36 mm. These outcomes translate directly into enhanced mastication, speech, airway function, and social participation [7,8,9,10,11,12].Consistent with our findings, Terletskyi et al. [63] emphasized that while patient-specific implants restore anatomy effectively, the preoperative functional baseline—particularly MIO—remains the most important determinant of long-term success.

### 4.2. Quality of Life Improvements

QoL enhancement emerged as a central outcome of TMJR, not merely a secondary benefit:

- Patients experienced improved diet, sleep, self-confidence, and return to normal life activities [7,10,11,12].
- Pediatric patients demonstrated parallel improvements in airway function, speech, and independence [11,22,31,32].
- Functional gains supported emotional and psychological recovery, reinforcing TMJR’s role in restoring holistic well-being.

These outcomes support TMJR as a life-improving procedure, particularly in refractory or disabling TMJ disease.

### 4.3. Prosthesis Type Comparison: Custom vs. Stock

Both prosthesis types delivered excellent results when used appropriately.

- Custom prostheses offered superior anatomic fit and slightly better MIO improvements in complex or revision cases [2,6,18,19].
- Stock prostheses achieved comparable pain relief and QoL outcomes in routine cases, with the advantages of reduced cost and immediate availability [8,20,21,22].

Meta-analyses found no significant difference in patient satisfaction or complication rates when prosthesis selection was individualized [20,23,24].

### 4.4. Pediatric TMJR Applications

Historically avoided, pediatric TMJR is now supported by promising medium-term outcomes.

- Children with severe ankylosis or joint destruction experienced restored function, airway expansion, improved chewing, and speech [11,22,31,32].
- No definitive evidence of growth inhibition has been reported in studies with follow-up of 2–8 years.
- However, long-term surveillance is essential. Pediatric TMJR patients may require prosthesis revision as they mature.

Expandable or growth-accommodating prosthetic designs remain a key unmet need [21,31,35].

### 4.5. Complication Management (Table 2)

When performed by experienced teams using standardized protocols, TMJR complication rates are comparable to or lower than those of orthopedic joint arthroplasty.

**Table 2:** Complication Management.

Complication	Prevention/Management
Heterotopic ossification (HO)	Autologous fat grafting (reduces incidence to <5%) [13,14]
Infection	Perioperative antibiotics, debridement, staged revision if needed [15,16]
Prosthesis failure	Revision surgery, often converting to custom device [16,32,34]
Chronic pain	Multimodal therapy targeting central sensitization and myofascial dysfunction [17,33]
Facial nerve injury	Surgical planning, nerve monitoring, meticulous dissection [46]

### 4.6. Technological Innovations

Technological advancements are transforming TMJR from mechanical replacement to digitally guided, biologically integrated treatment:

- CAD/CAM and 3D printing improve implant fit and reduce operative time [19,25,58].
- AR-guided surgery improves precision during placement and reduces surgical error [27,28].
- Biofilm-resistant materials (e.g., silver-ion, antibiotic-eluting coatings) are in development to lower infection risk [5,29,61].
- Smart implants with embedded sensors are being explored to monitor joint loading and detect early mechanical wear [62].

Tissue-engineered constructs offer a biologic alternative, especially for pediatric or reconstructive patients [29,30].

Together, these technologies are leading TMJR toward greater personalization, durability, and data-driven optimization.

### 4.7. Future Directions

To continue advancing TMJR care and research, we recommend the following:

1. Long-term outcome registries (e.g., UK TMJR database) to track prosthesis performance, complication rates, and QoL at 10–20 years [30].
2. Standardized outcome reporting using validated metrics (e.g., SF-36, TMJ-specific PROMs, VAS, MIO) across all centers.
3. Expandable prosthesis development for pediatric use and staged revision [33,35].
4. Multidisciplinary care models involving surgeons, engineers, pain specialists, and rehabilitation professionals.
5. Smart and regenerative implants to achieve biologic integration, sensor-based monitoring, and long-term adaptability [29,30,61].
6. As TMJR evolves, its success will depend not only on structural reconstruction but also on biologic integration, patient-centered design, and long-term functional outcomes.

## 5. Conclusions

Total temporomandibular joint replacement (TMJR) is a safe, effective, and transformative treatment for patients with end-stage TMJ pathology. This systematic review confirms that TMJR consistently results in significant improvements in pain relief, mandibular function—including increased maximum interincisal opening—and overall quality of life (QoL). Both custom and stock prostheses offer reliable outcomes, with custom designs showing advantages in complex or revision cases due to their superior anatomical fit. Pediatric patients also benefit from TMJR, particularly those with ankylosis or severe joint damage, and short- to medium-term follow-up studies have shown no definitive evidence of growth inhibition. However, ongoing surveillance remains necessary, and the development of expandable or growth-accommodating prosthetic designs is an important research priority. While complications such as heterotopic ossification, infection, and chronic postoperative pain can occur, most are preventable or manageable with appropriate surgical planning and perioperative care. Emerging technologies—including CAD/CAM planning, 3D printing, biofilm-resistant materials, and smart implants—are further enhancing the precision, durability, and personalization of TMJR. Future directions should focus on standardized outcome reporting, long-term registry data, and integration of biologically driven and sensor-enabled prosthetic designs to ensure continued improvement in patient-centered outcomes.

## Data Availability

All data used in this review are available from the cited peer-reviewed publications indexed in PubMed.
